# Allene oxide synthase, allene oxide cyclase and jasmonic acid levels in *Lotus japonicus* nodules

**DOI:** 10.1371/journal.pone.0190884

**Published:** 2018-01-05

**Authors:** Anna Zdyb, Marco G. Salgado, Kirill N. Demchenko, Wolfram G. Brenner, Małgorzata Płaszczyca, Michael Stumpe, Cornelia Herrfurth, Ivo Feussner, Katharina Pawlowski

**Affiliations:** 1 Department of Ecology, Environment and Plant Sciences, Stockholm University, Stockholm, Sweden; 2 Georg-August-University, Albrecht von Haller Institute for Plant Sciences, Department of Plant Biochemistry, Göttingen, Germany; 3 Komarov Botanical Institute, Russian Academy of Sciences, St. Petersburg, Russia; 4 Laboratory of Molecular and Cellular Biology, All-Russia Research Institute for Agricultural Microbiology, Laboratory of Molecular and Cellular Biology, St. Petersburg, Russia; 5 Institute of Biology/Applied Genetics, Dahlem Centre of Plant Sciences (DCPS), Freie Universität Berlin, Berlin, Germany; Estacion Experimental del Zaidin, SPAIN

## Abstract

Jasmonic acid (JA), its derivatives and its precursor *cis*-12-oxo phytodienoic acid (OPDA) form a group of phytohormones, the jasmonates, representing signal molecules involved in plant stress responses, in the defense against pathogens as well as in development. Elevated levels of JA have been shown to play a role in arbuscular mycorrhiza and in the induction of nitrogen-fixing root nodules. In this study, the gene families of two committed enzymes of the JA biosynthetic pathway, allene oxide synthase (AOS) and allene oxide cyclase (AOC), were characterized in the determinate nodule-forming model legume *Lotus japonicus* JA levels were to be analysed in the course of nodulation. Since in all *L*. *japonicus* organs examined, JA levels increased upon mechanical disturbance and wounding, an aeroponic culture system was established to allow for a quick harvest, followed by the analysis of JA levels in whole root and shoot systems. Nodulated plants were compared with non-nodulated plants grown on nitrate or ammonium as N source, respectively, over a five week-period. JA levels turned out to be more or less stable independently of the growth conditions. However, *L*. *japonicus* nodules formed on aeroponically grown plants often showed patches of cells with reduced bacteroid density, presumably a stress symptom. Immunolocalization using a heterologous antibody showed that the vascular systems of these nodules also seemed to contain less AOC protein than those of nodules of plants grown in perlite/vermiculite. Hence, aeroponically grown *L*. *japonicus* plants are likely to be habituated to stress which could have affected JA levels.

## Introduction

Jasmonates—jasmonic acid (JA) and its derivatives, such as its methyl ester (MeJA) and amino acid conjugates—are plant signalling compounds synthesized via the oxylipin pathway [1,2]. This pathway is initiated by the oxygenation of linoleic or α-linolenic acid by lipoxygenases (LOXs), leading to the formation of (9*S*)-hydroperoxy linoleic or α-linolenic acid (9-HPOD/9-HPOT) or (13*S*)-hydroperoxy linoleic or α-linolenic acid (13-HPOD/13-HPOT) [3]. Only the α-linolenic acid-derived product 13-HPOT can be converted to jasmonates [2]. The first committed step of JA biosynthesis is catalyzed by allene oxide synthase (AOS) which converts 13-HPOT to a highly reactive allene oxide, which in the second committed step is converted to *cis*-12-oxo phytodienoic acid (OPDA) by allene oxide cyclase (AOC). OPDA is converted to the corresponding 3-2(2’(Z)-pentenyl) cyclopentane-1-octanoic acid (OPC-8:0) stereoisomer by 12-oxophytodienoate reductase 3 (OPR3); OPC-8:0 is then converted to JA by three rounds of β-oxidation. This part of the JA biosynthetic pathway occurs in plastids, whereas the conversion of OPDA to JA takes place in peroxisomes [2].

JA signaling is involved in plant-pathogen interactions and wound signaling and has also been linked to developmental processes, such as root and flower development and the regulation of nitrogen storage [1,4,5]. It also plays a role in arbuscular mycorrhizal (AM) symbioses, where it is produced in the arbuscule-containing root cortical cells (reviewed by Hause and Schaarschmidt [6], Jung et al. [7]). Studies on *Medicago truncatula* have shown that a reduction in JA biosynthetic capacity interferes with the development of an arbuscular mycorrhizal symbiosis [8]. Interestingly, during the colonization of *Nicotiana attenuata* with the AM fungus *Rhizophagus irregularis*, root JA levels did not increase in response to AM colonization, and a defect in JA signaling had no effect on the interaction with the AM fungus [9]. Jasmonates have been implicated in the control of nodulation in root nodule symbioses [10,11,12,13], and also in nodule senescence [14]. However, in spite of the fact that the formation of the apparatus for jasmonate signaling seems to be induced early in *M*. *truncatula* nodule development [15], studies on *M*. *truncatula* plants with transgenic hairy roots with reduced JA biosynthetic capacity showed no effect on nodule frequency or -development [16].

*M*. *truncatula* forms indeterminate nodules; i.e., the cells in the inner tissue are arranged in a developmental gradient. For biochemical analyses of potential changes of jasmonate levels in the course of nodule development, determinate nodules are needed where the spatial developmental gradient is replaced by a temporal one and all infected cells in the inner tissue are more or less at the same developmental stage. The model legume *Lotus japonicus* forms determinate nodules. Thus, in order to analyse the role of jasmonates in the development of determinate nodules, we set about comparing the JA biosynthetic capacity by characterizing the enzymes involved in the two first committed steps of JA biosynthesis, AOS and AOC, in *L*. *japonicus*. Furthermore, the levels of JA were analysed in root and shoot systems of nodulated vs. non-nodulated *L*. *japonicus* in a time course experiment. In parallel, the cell-specific localization of AOC in nodules was followed.

## Materials and methods

### Plant and bacterial growth conditions

For transcriptional analyses, *Lotus japonicus* cv. Gifu plants were grown on a perlite/vermiculite mixture (1:1) wetted with ¼ strength Hoagland’s medium either supplemented with 10 m KNO_3_ or without N-source for nodulation [17]. Perlite and vermiculite were purchased from Weibull Trädgard AB (Hammenhog, Sweden). Greenhouse conditions were 150–300 μEm^-1^s^-1^ light intensity and ca. 23°C at 13 h light/11 h dark. For nodulation, plantlets were inoculated with *Mesorhizobium loti* strain TONO grown in TY medium [18], washed with and resuspended in double-distilled H_2_O, when they had developed primary leaves. Roots for transcriptional analyses were harvested from plants grown with KNO_3_ as N-source. Nodules were harvested three weeks after inoculation. For immunolocalization experiments, plants were watered with Fåhraeus medium without N source [19]; inoculation with strain TONO took place as described above, and nodules were harvested three weeks after inoculation.

For analyses of jasmonic acid, *L*. *japonicus* seeds were germinated on germination soil (S-jord, Weibull Trädgard AB) and after 5 weeks, plants were transferred to an aeroponic system (based on Cook et al. [20]) with medium according to Lullien et al. [21], and infected with *M*. *loti* strain TONO as described for perlite/vermiculite grown plants. Root and shoot systems were harvested at five time points, after 0, 7, 14, 21 and 28 days and shock-frozen in liquid nitrogen or fixed for immunolocalization experiments.

### Molecular cloning

Plant RNA was isolated as described by Demina et al. [22]. The First-Strand^®^ cDNA Synthesis Kit from GE Healthcare (Uppsala, Sweden) was used for reverse transcription. Three different DNA polymerases were used according to the manufacturer’s instructions: Taq (native, without BSA) from Fermentas (St. Leon-Rot, Germany), *PfuTurbo*^®^ DNA polymerase from Stratagene (La Jolla, CA, USA) and Platinum^®^ PCR SuperMix from Invitrogen (Lidingö, Sweden). Sequences of the PCR products were confirmed by Eurofins Genomics (Ebersberg, Germany).

*L*. *japonicus* cDNAs of interest (*aos1*, *aoc1*, *aoc2*) were cloned in the expression vectors pET-28a or pQE-30. For this purpose, *Ljaos1* was amplified with specific primers adding *Pst*I restriction sites (5’-ACTGCAGAGATGATGGCATCTTCTAC-3’ and 5’-ACTGCAGTTAAAAGCTTGCTCTCTTCAATG-3’. The resulting fragment was cloned in pGEM-T Easy. Afterwards, the *Ljaos1* cDNA was excised from this vector using *Pst*I and cloned in the *Pst*I site of the expression vector pQE-30, yielding pQE-30-*Ljaos1*. The orientation of the insert was determined using the asymmetric *Bam*HI restriction site in the *Ljaos1* cDNA. For the cloning of *Ljaoc1* in an expression vector, it was amplified with specific primers adding *Bam*HI restriction sites (5’-AAGGATCCCATCAACCACATCATTAGTTG-3’ and 5’-AAGGATCCATGGCCTCAATGGGTTCTC-3’). After cloning into pGEM-T Easy, the insert was excised using *Bam*HI and cloned into the *Bam*HI-digested pQE-30 vector. The orientation of the insert was determined using the asymmetric *Hin*dIII site in the *Ljaoc1* cDNA. For *Ljaoc2*, no cDNA clone containing the whole ORF was available; therefore, primers were designed based on genomic sequence information. *Ljaoc2* was cloned in the expression vector pET-28a after adding a 3’ *Bam*HI site and 5’ *XhoI* site (5’-GGATCCTCCTCTGAAACTGAGAG-3’ and 5’-CTCGAGGTTAGTGAAACCAGCAATGGT-3’). However, expression of this construct in *E*. *coli* Rosetta cells did not yield AOC enzyme activity. Such problems with the expression of *aoc* cDNAs in *E*. *coli* had been encountered earlier with the tomato gene [23,24]. Based on these earlier results, the DNA sequence encoding the 83 N-terminal amino acids, mostly comprising the transfer peptide, was removed from the 5’ end of the *Ljaoc2* cDNA by amplifying a truncated cDNA, again adding a 3’ *Bam*HI site and a 5’ *Xho*I site (5’-GGATCCTCCTCTGAAACTGAGAG-3’ and 5’-CTCGAGGTTAGTGAAACCAGCAATGGT-3’). The resulting fragment was cloned in pGEM-T Easy, and the insert was excised using *Bam*HI and *Xho*I and cloned in pET-28a.

### Protein isolation, protein gel electrophoresis, Western blot analysis and immunolocalization

Protein isolation, gel electrophoresis and Western blot analysis were performed as described by Zdyb et al. [16]. Immunolocalization was performed as described by Zdyb et al. [16]. Some sections were analysed using an LSM 510META Confocal Laser Scanning Microscope (Carl Zeiss, Jena, Germany). For visualization of AlexaFluor488, a 488 nm argon laser was used, and for Toluidine Blue a 561 nm laser line.

### JA determinations

Extraction of JA was performed as previously described for lipids, with some modifications [25]. Plant material (100 mg) was extracted with 0.75 mL of methanol containing 10 ng D_6_-JA (kindly provided by Otto Miersch, Halle/Saale, Germany) as internal standard. After vortexing, 2.5 mL of methyl-*tert*-butyl ether (MTBE) were added and the extract was shaken for 1 h at room temperature. For phase separation, 0.625 mL water was added. The mixture was incubated for 10 min at room temperature and centrifuged at 450 x g for 15 min. The upper phase was collected and the lower phase was re-extracted with 0.7 mL methanol and 1.3 mL MTBE as described above. The combined upper phases were dried under streaming nitrogen and resuspended in 100 μl of acetonitrile/water/acetic acid (20:80:0.1, v/v/v). The quantification of JA was subsequently performed based on HPLC-MS/MS analysis as described in Ibrahim et al. [26].

### Real-time reverse transcription-polymerase chain reaction (RT-PCR)

Total RNA was extracted from roots, nodules, stems, leaves, flowers and developing seed pods of *L*. *japonicus* using a modified version of the RNeasy Plant Mini Kit protocol (Qiagen, Hilden, Germany) combined with an on-column DNase treatment. Prior to cDNA synthesis, an additional DNase digestion was carried out using the Heat&Run Genomic DNA removal kit from ArcticZymes (Tromsø, Norway). 1 μg of total RNA per sample was reverse transcribed in a final volume of 20 ul following the instructions of the TATAA GrandScript cDNA synthesis kit (TATAA Biocenter, Göteborg, Sweden). cDNAs were diluted 10^−1^ and 2 ¼l were used as a template in 10 μl PCR reactions; these reactions were performed in 1x Maxima SYBR green (Thermo Fisher Scientific, Waltham, MA, USA) supplied with 300 nM of each primer in an Illumina^®^ Eco^™^ Real Time PCR platform. PCR conditions used were as follows: 10 min, 95°C for initial denaturation, and 45 cycles with a duration of 30 sec at 60°C followed by a melt dissociation curve. Controls for gDNA and primer dimer assessment were taken into account by the inclusion of water as a template, by RT-minus runs, and by melting dissociation curves analyses. In order to estimate and correct for primers efficiency, standard curves from a serial dilution of pooled cDNA were generated for each primer pair. Cq values correlating with the steady-state level of transcript abundance at the exponential phase were treated based on the ΔΔCt method using ubiquitin as an internal normalizer. For all organs, statistical analyses were carried out based on three biological replicates with two technical PCR repeats from which an unpaired 2-tail t-test was inferred using GenEx v. 5.4.1, MultiD Analyses (Askim, Sweden).

Primers were designed using Primer3 at the Primer-Blast NCBI server: 5’-CGGATTACAACATCCAGAAGG-3’ and 5’-GTAATGGTCTTACCAGTCAAGG-3’ for the housekeeping control (*L*. *japonicus* polyubiquitin; GenBank accession no. DQ249171), 5’-TGGTTTCGAGGTTGTTGG-3’ and 5’-GTGAGAGTAACAGCAGAACC-3’ for *Ljaos1*, 5’-AACCAACCTTGGGGGACAAG-3’ and 5’-TAACGCAAAAACAGCTCCGC-3’ for *Ljaos2*, 5’-TCAGCAACTTGTGTTCCC-3’ and 5’-GAAGGATCAACAGGCTTCC-3’ for *Ljaoc1*, 5’- CAGAGAAGAATGGTGACAGG and 5’-TCCTCATAGGTCAGGTATGG-3’ for *Ljaoc2* and 5’- GGTCCTTACCTGACCTATGA -3’ and 5’- GCTTGACCTGACCATACAC -3’ for *Ljaoc3*.

### Analyses of enzyme activities

The assays were performed using 13-HPOT ((13*S*)-hydroperoxy-(9*Z*,11*E*,15*Z*)-octadecadienoic acid), which was obtained as described previously [27]. For the enzyme activity assays, *Ljaos1*, *Ljaoc1*, and *Ljaoc2* cDNAs were expressed in *E*. *coli* in the presence of the appropriate antibiotic at 16°C for 24 h after induction with 0.1 mM IPTG. Expression of the *Ljaos1* cDNA was performed in *E*. *coli* strain SG13009, chosen because it is well suited for growth at low temperatures (16°C), which allows overexpression of membrane-associated proteins more effectively. The assays were carried out as described before with small modifications [28]. The resulting products were extracted as previously described [29] and analyzed on reverse phase HPLC [30].

## Results and discussion

### Identification of allene oxide synthase (*aos*) and allene oxide cyclase (*aoc*) genes from *Lotus japonicus*

In order to analyse JA biosynthesis in roots and nodules of *L*. *japonicus* grown under different conditions, two enzymes of the JA biosynthetic pathways were chosen for characterization, AOS and AOC. The first step was to identify the members of the *aos* and *aoc* gene families in *L*. *japonicus* using publicly available sequence information as well as information from the *L*. *japonicus* sequencing project at http://www.kazusa.or.jp/lotus/. For this purpose, the corresponding gene sequences from *Arabidopsis thaliana* (*aos* [31]; *aoc* [32]) were used to identify the corresponding genes from *L*. *japonicus*. Our results showed that in *L*. *japonicus* AOS was encoded by two genes, which were named *Ljaos1* and *Ljaos2*. AOC enzymes were encoded by a small gene family with three members, named *Ljaoc1*, *Ljaoc2* and *Ljaoc3*.

Sequence analysis revealed that the *Ljaos1* cDNA (GenBank accession number AB600747.1) was 1918 bp in length, containing an open reading frame (ORF) of 1587 bp. The molecular weight of the AOS1 protein was 59.3 kDa with a predicted isoelectric point (pI) of 8.41 according to Kozlowski [33]. Further bioinformatic analysis using the ChloroP 1.1 [34] and the Plant-mPLoc server [35] revealed that the LjAOS1 protein contained a putative N-terminal plastid targeting sequence of 37 amino acids. The *Ljaos2* cDNA (Lj1g3v1604250.1 on www.kazusa.org and lotus.au.dk) was 2822 bp in length with an ORF of 1599 bp. The molecular weight of AOS2 was 60.2 kDa with a predicted pI of 8.39. Bioinformatic analysis using ChloroP 1.1 and Plant-mPLoc showed that LjAOS2 was a plastidic protein with a targeting sequence of 57 amino acids.

It should be noted that while *Arabidopsis thaliana* as well as *Medicago truncatula* contain a single *AOS* gene [36,30], larger gene families are common in legumes. E.g., analysis of legume genomes available at https://legumeinfo.org/ shows that narrow-leafed lupine (*Lupinus angustifolius* L.) and red clover (*Trifolium pratense* L.) have *AOS* gene families with seven members, cowpea (*Vigna unguiculata* (L.) Walp.) has six *AOS* genes and the two diploid ancestors of peanut (*Arachis duranensis* Krapov. & W.C. Gregory and *Arachis ipaensis* Krapov. & W.C. Gregory) have six and five *AOS* genes, respectively.

Analysis of *aoc* sequences revealed that the *Ljaoc1* cDNA (GenBank accession number BT141471) was 893 bp in length and contained an ORF of 771 bp encoding a 28 kDa protein of 256 amino acids with a calculated pI of 8.49 and a putative N-terminal plastidic targeting sequence of 53 amino acids. The *Ljaoc2* cDNA (Kazusa accession number chr1.CM0012.1230.r2.m) was 986 bp long and contained a 768 bp ORF encoding a 28 kDa protein of 255 amino acids with a calculated pI of 8.11 and a putative N-terminal plastid targeting sequence of 71 amino acids. *Ljaoc3*, a 836 bp cDNA (Genbank BT138810.1), contained a 747 bp ORF encoding a 27.25 kDa protein of 248 amino acids with a calculated pI of 7.87 and a putative N-terminal plastid targeting sequence of 66 amino acids.

While *A*. *thaliana* has an *AOC* gene family with four members [32], *M*. *truncatula* has only two *AOC* genes (see [8] for the gene encoding the 257 amino acid AOC1, GenBank accession XP_013451276.1; the genome sequence revealed a second gene encoding a 234 aa isoform, GenBank accession KEH25317.1). Based on https://legumeinfo.org/genomes, red clover has two *AOC* genes and cowpea has three, while narrow-leafed lupine has seven and the two progenitors of peanut have five (*A*. *duraensis*) and four (*A*. *ipaensis*), respectively. In short, with three *AOC* genes, *L*. *japonicius* is in the normal range for legumes.

### Transcript levels of *Ljaos1* and *Ljaos2* as well as *Ljaoc1*, *Ljaoc2* and *Ljaco3* in different organs

**Fig 1:**
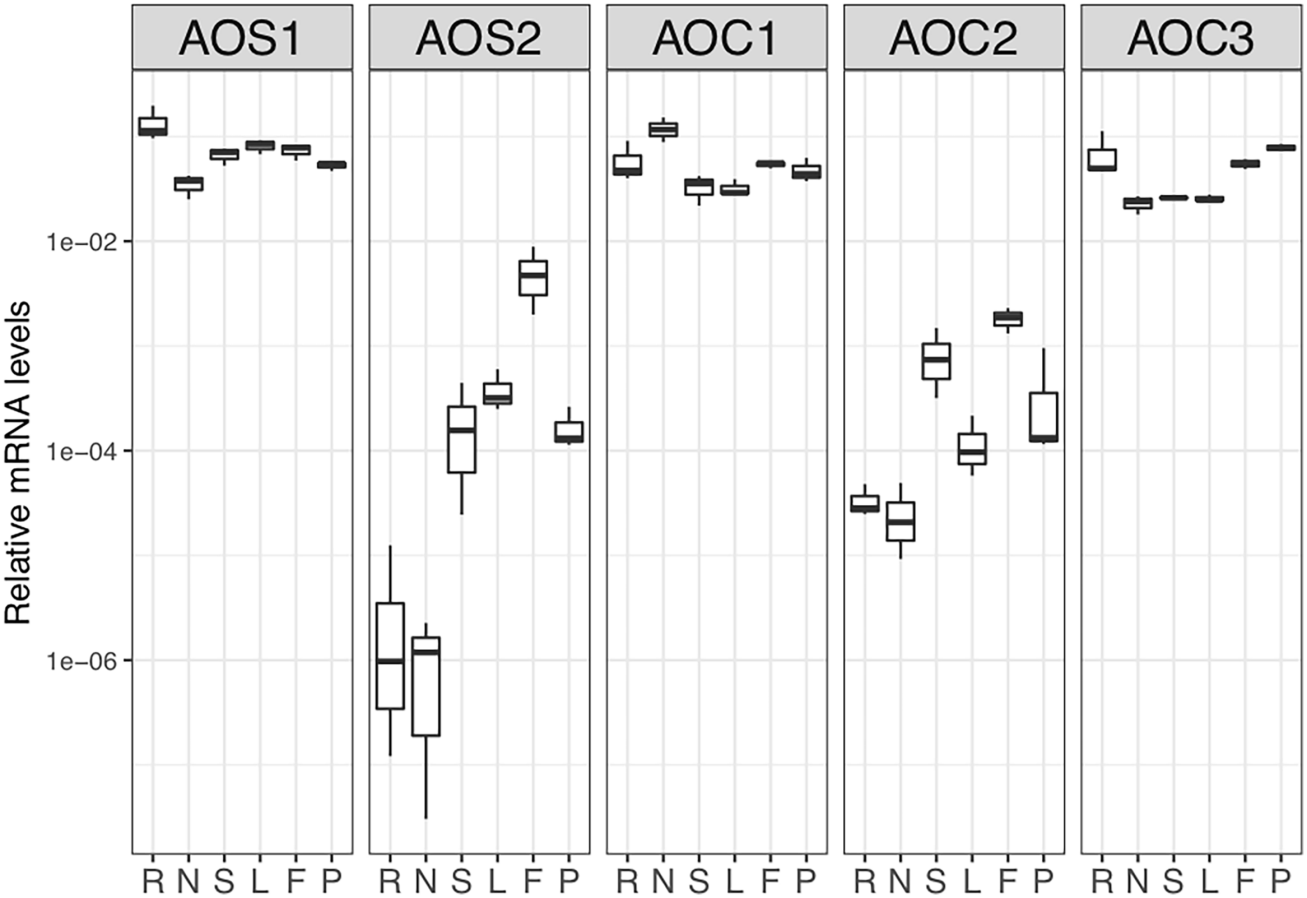
Expression analysis of *Ljaos1*, *Ljaos2*, *Ljaoc1*, *Ljaoc2* and *Ljaoc3* genes in roots (R), nodules (N), stems (S), leaves (L), flowers (F) and immature pods (P) of *L*. *japonicus* cv. Gifu using real time RT-PCR. *L*. *japonicus* ubiquitin was used as housekeeping control. Three biological replicates were used. The data are presented as box plot. The boxes show the interquartile range, and bars indicate data points below the first and above the third quartile. Lines in the boxes mark the median value.

In order to analyse organ-specific expression of *Ljaos1* and *Ljaos2* and of the three members of the *L*. *japonicus aoc* gene family, levels of each transcript were analysed in different organs of *L*. *japonicus* roots, nodules, stem, leaves, flowers and immature pods using quantitative real time RT-qPCR. *Ljaos1* transcripts were present in all organs examined at similar levels (Fig 1), while *Ljaos2* transcript levels were much lower than those of *Ljaos1*. Substantial intra-tissue variation was observed in *Ljaos2* transcript levels in both roots and nodules, in contrast with the other genes examined. This suggests the existence of regulatory mechanisms other than tissue specificity for *Ljaos2* expression. From the members of the *aoc* gene family, *Ljaoc1* and *Ljaoc3* showed the highest expression levels in all organs. *Ljaoc2* showed the lowest expression levels of all *aoc* genes*;* in particular its expression levels in roots and nodules were very low. None of the transcripts was induced significantly in nodules compared to roots or *vice versa*. In all organs examined, *AOS1* and *AOC1/AOC3* seemed to play the major role. Interestingly, although JA is required for reproductive development in many plant species [5], when compared with the *A*. *thaliana AOC* gene family, only *AOC2* was significantly induced in flowers compared to roots, and *AOC2* transcript levels in flowers were still an order of magnitude lower than those of *AOC1/3*. This could mean either that *AOC* expression levels in roots of *L*. *japoncius* are higher than in Brassicaceae [32] and Solanaceae [37], or that *AOC* expression levels in flowers of *L*. *japonicus* are lower than in flowers of other plants. The fact that MtAOC1 protein levels are similar in roots and flowers of *M*. *truncatula* [8] would seem to imply that the phenomenon is not restricted to *L*. *japonicus* and might be common for legumes.

### Biochemical characterization of LjAOS1

**Fig 2:**
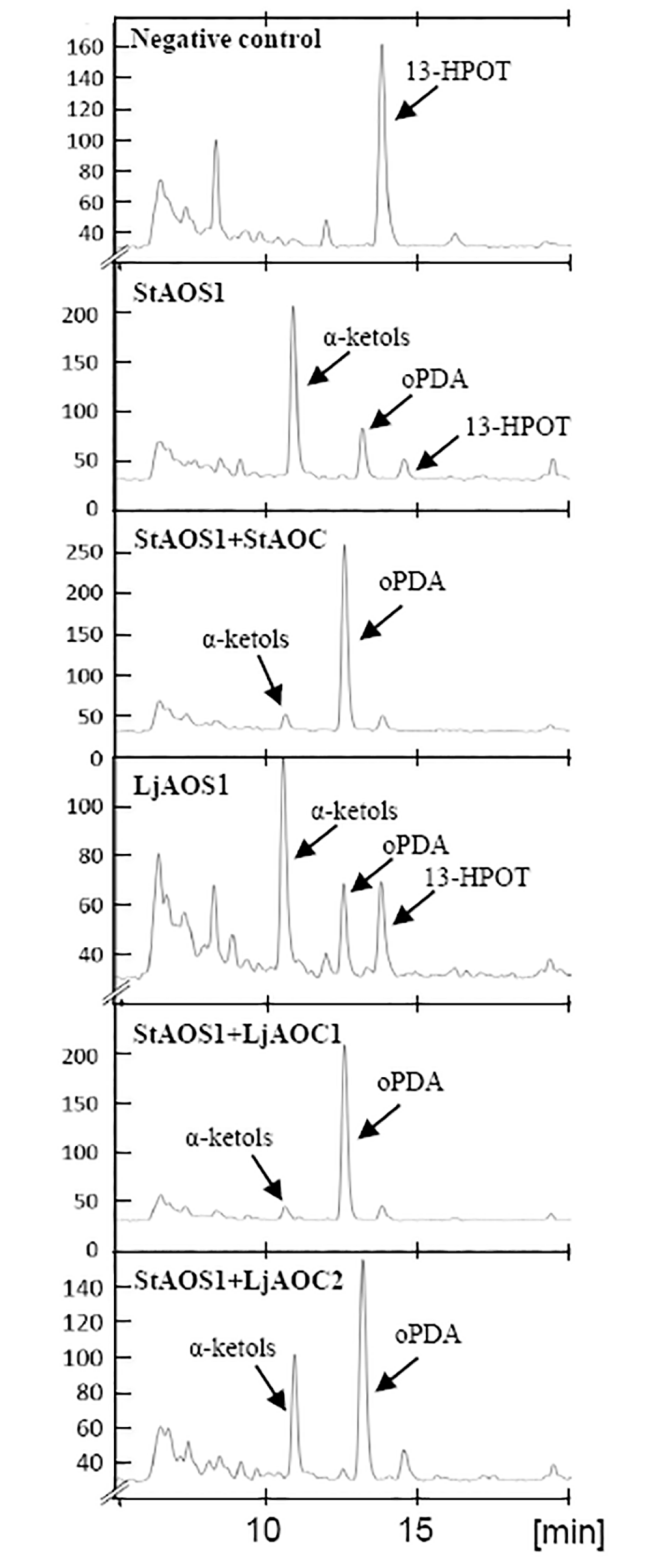
Enzyme activity assays. Enzymatic conversion of [1-^14^C]-13-HPOT was analyzed by radio-HPLC. *Solanum tuberosum* AOS1 and AOC (Stumpe et al., 2006) were used as positive controls. The chromatogram shows α-ketol as well as OPDA as LjAOS1 reaction products, and OPDA as the only reaction product for LjAOC1 and LjAOC2 which were used in combination with *S*. *tuberosum* AOS1. mAU, milli absorption units. One representative of three independent experiments is shown.

Since *Ljaos1* expression levels were so much higher than those of *Ljaos2*, *Ljaos1* was chosen for biochemical characterization of the encoded enzyme. For this purpose, the *Ljaos1* cDNA was expressed in *E*. *coli* strain SG13009. In the assay performed on the cell lysate, [1-^14^C]-13-HPOT was used as a substrate. Products were extracted and analyzed by radio-HPLC. Since the resulting allene oxide is very unstable, its hydrolysis product, the α-ketol, was detected as specific reaction product for LjAOS1. As a positive control, a cDNA expression clone of the previously described *Solanum tuberosum* AOS1 was used [28]. In each case, three independent colonies were tested and were active in the enzyme assay. The results of one representative clone each are shown in Fig 2.

Plant AOS enzymes belong to the CYP74 protein family, a group of cytochrome P-450s that are specialized for the metabolism of fatty acid hydroperoxides [38]. Three different types of AOS enzymes are known. The first two types can use either (13*S*)-hydroperoxides or (9*S*)-hydroperoxides as substrates (subfamilies CYP74A and CYP74C, respectively), while the third type can use both of them (CYP74B; reviewed by Stumpe and Feussner [38]). To further classify LjAOS1 and LjAOS2, a phylogenetic analysis was performed using various plant CYP74 protein sequences. Multiple alignments of the sequences were performed using ClustalX and a phylogram was constructed using the PHYLIP program package. The phylogram showed that LjAOS1 and LjAOS2 both belong to the CYP74A subfamily whose members are specific for (13*S*)-hydroxyperoxides (S1 Fig).

### Biochemical characterization of LjAOC1 and LjAOC2

The transcriptional analysis had shown that *Ljaoc1* was the dominant *aoc* gene in nodules of *L*. *japonicus*, followed by *Ljaoc3*, while *Ljaoc2* was expressed at the lowest levels. LjAOC3 showed similar levels of amino acid identity (69.5% vs. 70.2%) with LjAOC1 and LjAOC2, respectively, while there were only 56.1% amino acid identify between LjAOC1 and LjAOC2. Therefore, we chose LjAOC1 and LjAOC2 for biochemical characterization.

After the expression of *Ljaoc1* and *Ljaoc2* in *E*. *coli* SG13009, a coupled enzyme activity assay was performed. As a positive control, an expression vector with a cDNA encoding AOC1 of *S*. *tuberosum* [30] was used. The lysates containing AOC were mixed with lysates containing the AOS1 from *S*. *tuberosum* [28] and [1-^14^C]-13-HPOT was added, which was first transformed into allene oxide, the substrate of AOCs, by StAOS1. Both LjAOC enzymes were tested three times independently; Fig 2 shows one representative result for each construct. Both AOCs, the full-length LjAOC1 and the truncated version of LjAOC2, were enzymatically active, based on the fact that they catalyzed the synthesis of OPDA.

### Amounts of AOC protein in roots *vs*. nodules

**Fig 3:**
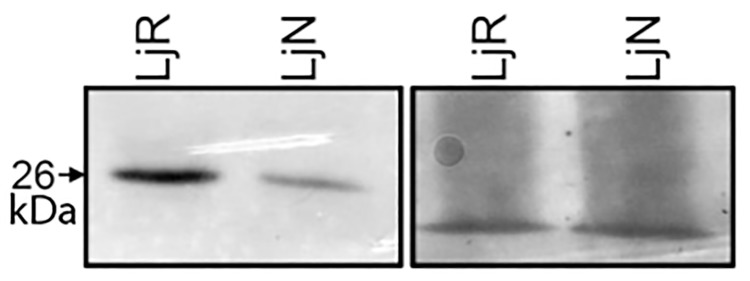
AOC protein levels in roots and nodules. The left panel shows a Western blot from root (LjR) and nodule (LjN) proteins from *L*. *japonicus*, developed with an antibody against tomato AOC. The right panel shows a 12% SDS-polyacrylamide gel with the same amount of protein loaded, stained with Fast Green. AOC proteins are 26 kDa in size. One representative example of three independent experiments is shown.

Since a specific antibody for AOS was not available, we tested only the abundance of AOC protein. Using the anti-tomato AOC antibody [26], which should recognize all isoforms of *L*. *japonicus* AOC, relative amounts of AOC protein in roots and nodules were analyzed by Western blotting. Levels of AOC protein detected in nodules were significantly lower than those detected in roots (Fig 3). This was interesting since according to the transcriptional analysis (Fig 1), the combined transcript levels of *Ljaoc1*, *Ljaoc2* and *Ljaoc3* in nodules were in the same range as in roots. It has to be concluded that either not all *Ljaoc* transcripts are translated at the same efficiency in all organs/cell types, or that AOC protein is more stable in roots than in nodules, or that the difference is due to the fact that the nodule extract contained increased amounts of membrane proteins due to the peribacteroid membranes, and/or due to the presence of rhizobial proteins.

### JA levels in nodulated *vs*. non-nodulated roots of *L*. *japonicus*

*L*. *japonicus* with its determinate nodules was used as a model system to investigate the JA levels in roots compared to nodules. Changes in JA levels during nodule development were also tested and compared to JA levels in non-nodulated *L*. *japonicus* plants grown under different nitrogen conditions.

Previous results had shown the effects of mechanical disturbance on JA levels in roots and nodules [16,39]. This phenomenon was also examined for *L*. *japonicus*; here, leaves were included in the comparison. Nodules and roots were harvested from an individual plant in two steps 30 min apart. The first harvest represented the undisturbed sample and the second step yielded the mechanically disturbed (‘shaken’) control. The results showed that levels of JA and OPDA in *L*. *japonicus* roots, nodules and leaves increased in response to mechanical disturbance and wounding (data not shown).

**Fig 4:**
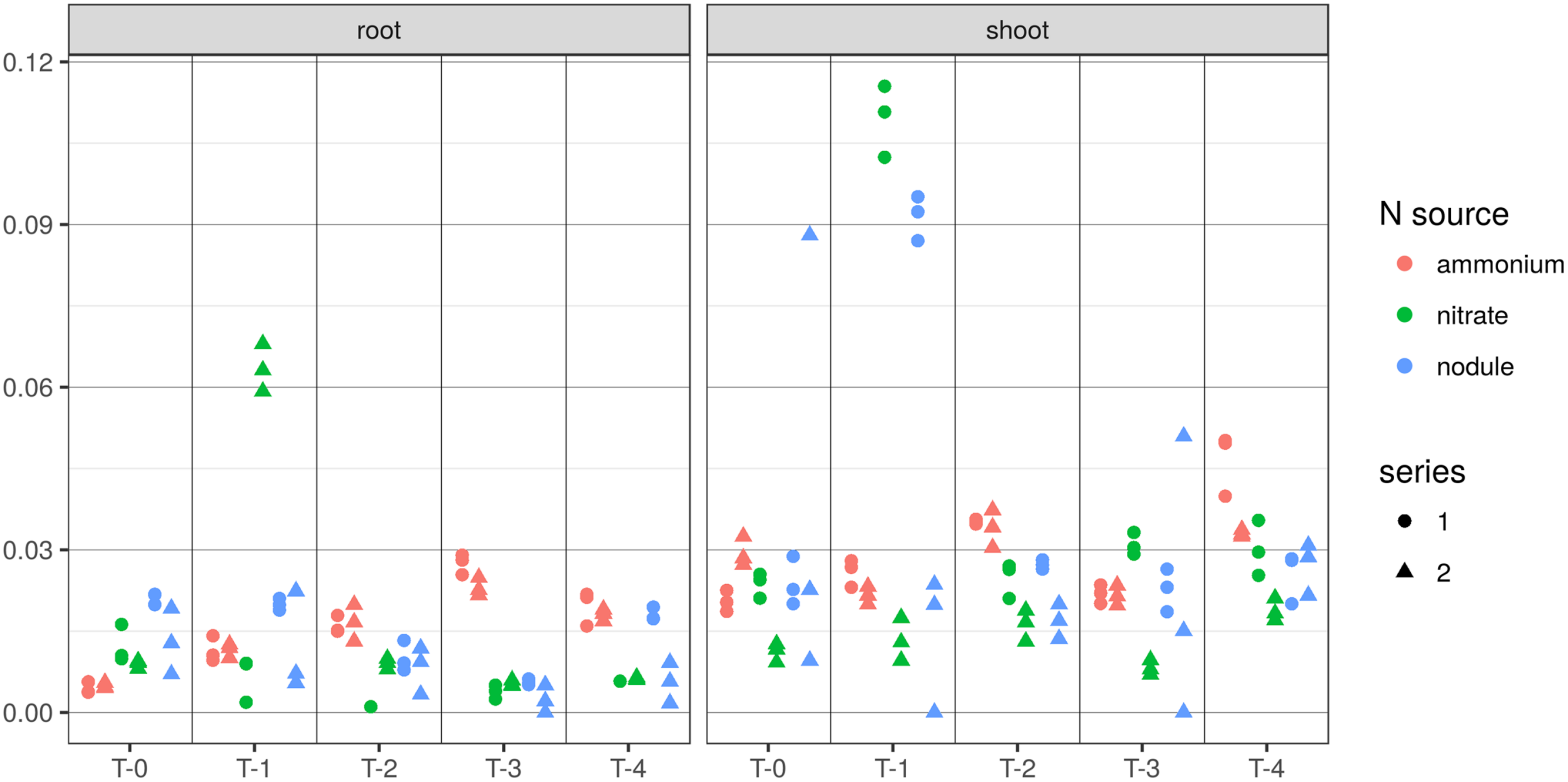
JA levels [nmol/g FW] measured in root and shoot systems of *L*. *japonicus* plants grown with a nitrogen source or with rhizobia. Results show six independent plant series grown under three different growth conditions, i.e. two series per growth condition. T-0 marks the beginning of the experiments when 5-week-old plants were transferred from soil to the aeroponic tank. Subsequent samples were collected in weekly intervals: T-1 after one week, T-2 after two weeks, T-3 after three weeks, T-4 after four weeks. In the case of the series grown without nitrogen, infection with *M*. *loti* strain TONO took place at T-0. At least five plants were harvested per time point; three technical replicates were analysed based on their combined root- or shoot systems, respectively. The data were evaluated using R [46] and plotted using ggplot2 (version 2.2.1, [47]). The analytical error is too large to allow the detection of statistically significant differences (Mann Whitney U) between the series.

To avoid the effect of mechanical disturbance on JA levels in further studies, a growth system had to be used, where root systems did not have to be cleaned during harvesting. Therefore, the aeroponic gowth system was chosen. For the same reason, separation of nodules from roots was not an option. Instead, each plant was cut at the hypocotyl so that root and shoot system could be frozen immediately in liquid nitrogen. This reduced the duration of handling of each individual plant to a matter of seconds. Five week old plants of *L*. *japonicus* were transferred from soil to the aeroponic system, and samples were collected at five time points, after 0, 7, 14, 21 and 28 days. Infection with rhizobia took place on the day of transfer. At least five plants were harvested per time point. Non-nodulated plants grown on two different sources of nitrogen, potassium nitrate and ammonia, respectively, were examined as well. Two series were examined for each growth condition. The results are presented in Fig 4. No significant correlation between JA levels and the stage of nodule development was observed in any of the experiments. There were also no significant differences in JA levels between the plants grown on nitrate or ammonium, and the nodulated ones. The JA values at 7 dpi (T-1) contain two outliers; in one of the two series, roots of nitrate grown plant have very high JA levels, while in the other series, shoots of nodulated plants show very high JA levels (Fig 4). Since in both cases, these very high values were restricted to one series, they might be explained by anthropogenic mechanical disturbance.

### Distribution of AOC in *L*. *japonicus* nodules

**Fig 5:**
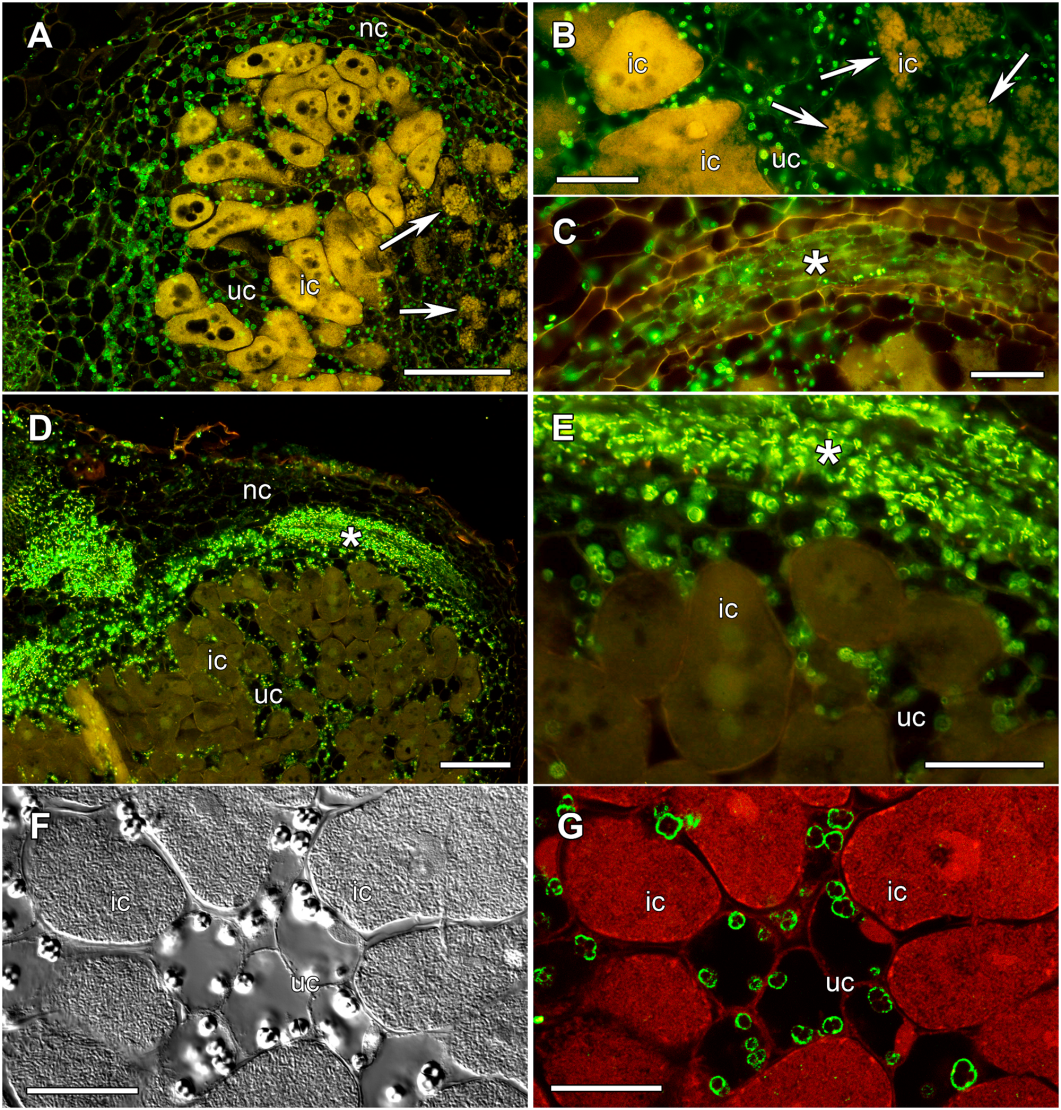
Immunolocalization of *L*. *japonicus* AOC protein in longitudinal sections of *L*. *japonicus* nodules. The nodules come from plants from two different growth systems; (A-C, F-G) aeroponic culture, (D-E) perlite/vermiculite-grown plants (in both cases, nodules were harvest 3 weeks after inoculation). Panels (A-E) show conventional wide-field fluorescent microscopy images where the fluorescence of the secondary antibody labeling AOC is visible in green. The image in panel (F) was taken with differential interference contrast. The image in panel (G) was taken on a confocal laser scanning microscope and shows immunolabeled AOC in green, while toluidine blue-stained bacteria and nuclei are shown in red. Labels: ic, infected cells; nc, nodule cortex; uc, uninfected cells of the inner tissue. The nodule vascular bundles are labeled with asterisks. The arrows in panels (A) and (B) points at infected cells with low bacteroid density. Size bars denote (A,D) 100 μm, (B,C,E) 50 μm, and (F,G) 20 μm.

The distribution of AOC protein in nodules of *L*. *japonicus* was analyzed using immunolocalization using the same heterologous antibody that had been used for Western blot analysis. In the initial experiment, the distribution of AOC was studied during nodule development. Nodules were harvested form *L*. *japonicus* grown in the aeroponic system at different time points, always a week apart. Harvesting point T-0 was the day of inoculation with *Mesorhizobium loti* strain TONO. There were no differences in AOC localization between the time points (data not shown). In nodules of all ages, AOC protein was present in the nodule cortex, nodule vascular parenchyma, and the uninfected cells of the inner tissue (Fig 5A–5E). Detailed observation under a confocal laser scanning microscope revealed that AOC was localized in the plastidic stroma (Fig 5F–5G).

An interesting phenomenon was observed regarding the structure of infected cells of nodules grown in aeroponic culture. Some infected cells showed a reduced density of bacteroids (see arrows in Fig 5A and 5B), presumably due to degradation of the bacteroids. This phenomenon was observed already in two-week-old nodules. If this phenomenon were related to early nodule senescence, cells with reduced bacteroid density would be expected to appear with increased frequency in older nodules. However, the phenomenon was found at similar frequency in two-week-old, three-week-old and four-week-old nodules, although only 6–8 nodules per time point were analyzed in detail. Developmental as well as stress-induced nodule senescence is related to increased nitric oxide levels [40]. Apart from being a side effect of bacteroid degradation during senescence, so far the phenomenon of reduced bacteroid density had been described only for *L*. *japonicus* nodules induced by a Fix^**-**^ mutant of *M*. *loti* [41]. Hence, altogether it seems likely that this observed low bacteroid density is due to bacteroid degradation caused by stress due to the growth conditions.

To test this hypothesis, AOC immunolocalization experiments were performed on nodules of plants grown in a perlite/vermiculite mixture wetted with ¼ strength Hoagland’s medium. Thus, the two growth systems used not only differed in substrate, but also regarding the salt concentrations in the growth medium. This, however, was unavoidable since aeroponic culture, with water droplets drying on the root surface, poses peculiar requirements with regard to the concentration of nutrients in the growth medium. Three week old nodules were harvested from perlite/vermiculite-grown plants and immunolocalization was performed using the same protocol as before, comparing sections from aeroponically grown and from perlite/vermiculite-grown nodules. The results confirmed the localization of AOC in the nodule cortex, uninfected cells of the inner tissue and nodule vascular parenchyma in nodules of perlite/vermiculite grown plants (Fig 5D–5E). However, when comparing AOC fluorescence in the uninfected cells of the inner tissue with AOC fluorescence in the vascular system, there was a striking difference between nodules from the two different growth systems. When AOC protein levels were compared in nodules from perlite/vermiculite grown plants vs. those of aeroponically grown plants, the difference in distribution was striking: in nodules from perlite/vermiculite grown plants, the highest levels of AOC were in the nodule vascular tissue. In nodules from aeroponically grown plant, the highest levels of AOC were in the uninfected cells of the inner tissue (compare Fig 5C with 5E). This could be explained by a reduction of AOC levels in the vascular system of aeroponically grown plants. No infected cells from perlite/vermiculite grown nodules were found to exhibit reduced bacteroid density. Hence, the occasional occurrence of reduced bacteroid density in infected cells, as well as the reduced amount of AOC in the nodule vascular system, was a phenomenon related to aeroponic cultivation of *L*. *japonicus*. Similar reduced bacteroid density has been published for ineffective nodules induced by *Rhizobium etli* on *L*. *japonicus* showing early senescence [42] and for senescent *L*. *japonicus* nodules formed by *sen1* mutant plants [43]; however, in those nodules the reduced bacteroid density was consistent in all infected cells. This similarity leads to the suggestion that the areas with reduced bacteroid density were senescent.

Research on *Phaseolus vulgaris* has shown that accumulation of salts on the surface or roots of plants in aeroponic culture can lead to osmotic stress [44], and osmotic stress can lead to bacteroid degradation like is taking place during senescence [45]. Thus, it is likely that in spite of the fact that the close relative of *L*. *japonicus*, *M*. *truncatula* does not have growth problems in the aeroponic system described [20], *L*. *japonicus* was stressed. While this stress might have initially led to an increase in JA levels, it is plausible that it would later have led to habituation. This could have caused reduced levels of JA in the plants, and maybe also led to a reduction of inducible JA biosynthesis. The latter would be in agreement with results from this study that nodules from plants grown in the aeroponic system showed much less AOC protein in their vascular system than those from perlite/vermiculite-grown plants. In this context, it is interesting that *M*. *truncatula* plants exposed to mechanostimulation three times per week displayed a shoot growth phenotype commensurate with increased JA levels, but while their shoots and roots showed enhanced levels of *MtAOC1* transcription, they did not contain increased JA levels [39]. At any rate, the fact that the nodules of aeroponically grown *L*. *japonicus* plants display a stress-related phenotype means that the observed levels of a stress-related phytohormone, JA, might well be affected by factors other than nodule development.

In summary, because of the side effects of mechanical disturbance on JA biosynthesis, aeroponic or hydroponic culture was required to enable quick harvesting of *L*. *japonicus* root systems for the determination of JA levels. However, although the aeroponic culture system used in this study reliably allowed good nodulation and did not cause any obvious growth defects, detailed analyses suggested that it caused low level stress on the plants which affected infection density and might have affected JA production.

## Conclusions

The *aos* and *aoc* gene families of the model legume *Lotus japonicus* were characterized. Enzyme activities of LjAOS1 and of two members of the LjAOC family, LjAOC1 and LjAOC2, were confirmed using expression in *E*. *coli*. LjAOC proteins were localized in *L*. *japonicus* nodules at different points of development using a heterologous antibody. Like in *Medicago truncatula* nodules [16], LjAOC proteins were present exclusively in the plastidic stroma of uninfected nodule cell types, namely in the nodule cortex, nodule vascular parenchyma, and the uninfected cells of the inner tissue. Changes in JA levels in the course of nodule development were analysed using an aeroponic growth system. No significant differences were found either between JA levels in root and shoot systems, respectively, under different forms of nitrogen supply, or over the course of nodule development. However, detailed analyses of nodules formed in aeroponic culture suggested that this growth system was sub-optimal for *L*. *japonicus*. While nodules formed in aeroponic culture were macroscopically indistinguishable from nodules formed on the roots of perlite/vermiculite-grown plants, nodule development and relative amounts of LjAOC protein in uninfected cells of the inner tissue *vs*. the nodule vascular system were affected in the aeroponic system.

## Supporting information

S1 Fig Phylogenetic tree of the *CYP74* enzyme family. (PDF)
