# Supplementary material for: Allene oxide synthase, allene oxide cyclase and jasmonic acid levels in Lotus japonicus nodules
Source: PLoS One. 2018 Jan 5;13(1):e0190884. doi: 10.1371/journal.pone.0190884 (PMC5755929; doi:10.1371/journal.pone.0190884)
Supplement: S1 Fig — (PDF) [file pone.0190884.s001.pdf]

S1 Fig. Phylogenetic tree of the *CYP74* enzyme family. The family encompasses allene oxide synthases (AOS; CYP74A and C), hydroperoxide lyases (HPL; CYP74B and C) and divinylether synthases (DES; CYP74B, D, H and Q). *L. japonicus* AOS1 and AOS2 belong to the 13-AOS group (based on Stumpe and Feussner [38]). Accession numbers of the sequences used in the analysis: *A. thaliana* AtAOS, CAA63266; AtHPL, AAC69871; *Allium sativum* AsDES, CAI30435; *Citrus sinensis* CiAOS, AAO72741; *Cucumis melo* CmAOS, AAM66138; CmHPL, AAK54282; *Cucumis sativus* CsHPL1, AAF64041; CsHPL2, AF229812; *Hordeum vulgare* HvAOS1, CAB86384; HvAOS2, CAB86383; HvHPL, CAC82980; *Linum usitatissimum* LuAOS, AAA03353; *Musa acuminata* MaHPL, CAB39331; *Medicago sativa* MsHPL1, CAB54847; MsHPL2, CAB54848; MsHPL3, CAB54849; *M. truncatula* MtAOS, CAC86897; MtHPL1 CAC86898; MtHPL2, CAC86899; *Nicotiana attenuata* NaAOS2, CAC82911; NaHPL, CAC91565; *Nicotiana tabacum* NtDES, AAL40900; *Oryza sativa* OsAOS1, AAL38184; OsAOS2, AAP50956; *Parthenium argentatum* PaAOS, CAA55025; *Prunus dulcis* PdHPL, CAE18065; *Psidium guajava* PgHPL, AAK15070; *Physcomitrella patens* PpAOS, CAC86919; PpHPL, CAC86920; *Solanum lycopersicum* SlAOS1, CAB88032; SlAOS2, AAF67141; SlAOS3, AAN76867; SlCYP74, AAL86702; SlDES, AAG42261; SlHPL, AAF67142; *Capsicum annuum* CaHPL2, AAA97465; *Solanum tuberosum* StHPL, CAC44040; StAOS2, ABD15175; StAOS3, CAI30876; StDES, CAC28152; *Triticum aestivum* TaAOS, AAO43440; *Zea mays* ZmHPL, from patent WO00/22145; *Selaginella moellendorfii* SmCYP74M1 (DES), XP_002979266; SmCYP74M3 (DES), XP_002964012.

The evolutionary history was inferred using the UPGMA method [S1]. The optimal tree with the sum of branch length = 9.80131868 is shown. The tree is drawn to scale, with branch lengths in the same units as those of the evolutionary distances used to infer the phylogenetic tree. The evolutionary distances were computed using the Poisson correction method [S2] and are in the units of the number of amino acid substitutions per site. The analysis involved 46 amino acid sequences. All ambiguous positions were removed for each sequence pair. There were a total of 595 positions in the final dataset. Evolutionary analyses were conducted in MEGA7 [S3].

It should be noted that the phylogeny of CYP74 genes in Bryophyta and Lycopodiophyta is not completely understood yet [S4].

S1. Sneath PHA, Sokal RR (1973). Numerical Taxonomy. Freeman, San Francisco.

S2. Felsenstein J (1985). Confidence limits on phylogenies: An approach using the bootstrap. Evolution 39:783–791.

S3. Kumar S, Stecher G, Tamura K (2016). MEGA7: Molecular Evolutionary Genetics Analysis version 7.0 for bigger datasets. Molecular Biology and Evolution 33:1870–1874.

S4. Gorina SS, Toporkova YY, Mukhtarova LS, Smirnova EO, Chechetkin IR, Khairutdinov BI, Gogolev YV, Grechkin AN (2016). Oxylipin biosynthesis in spikemoss *Selaginella moellendorffii:* Molecular cloning and identification of divinyl ether synthases CYP74M1 and CYP74M3. Biochim Biophys Acta 1861:301–309.

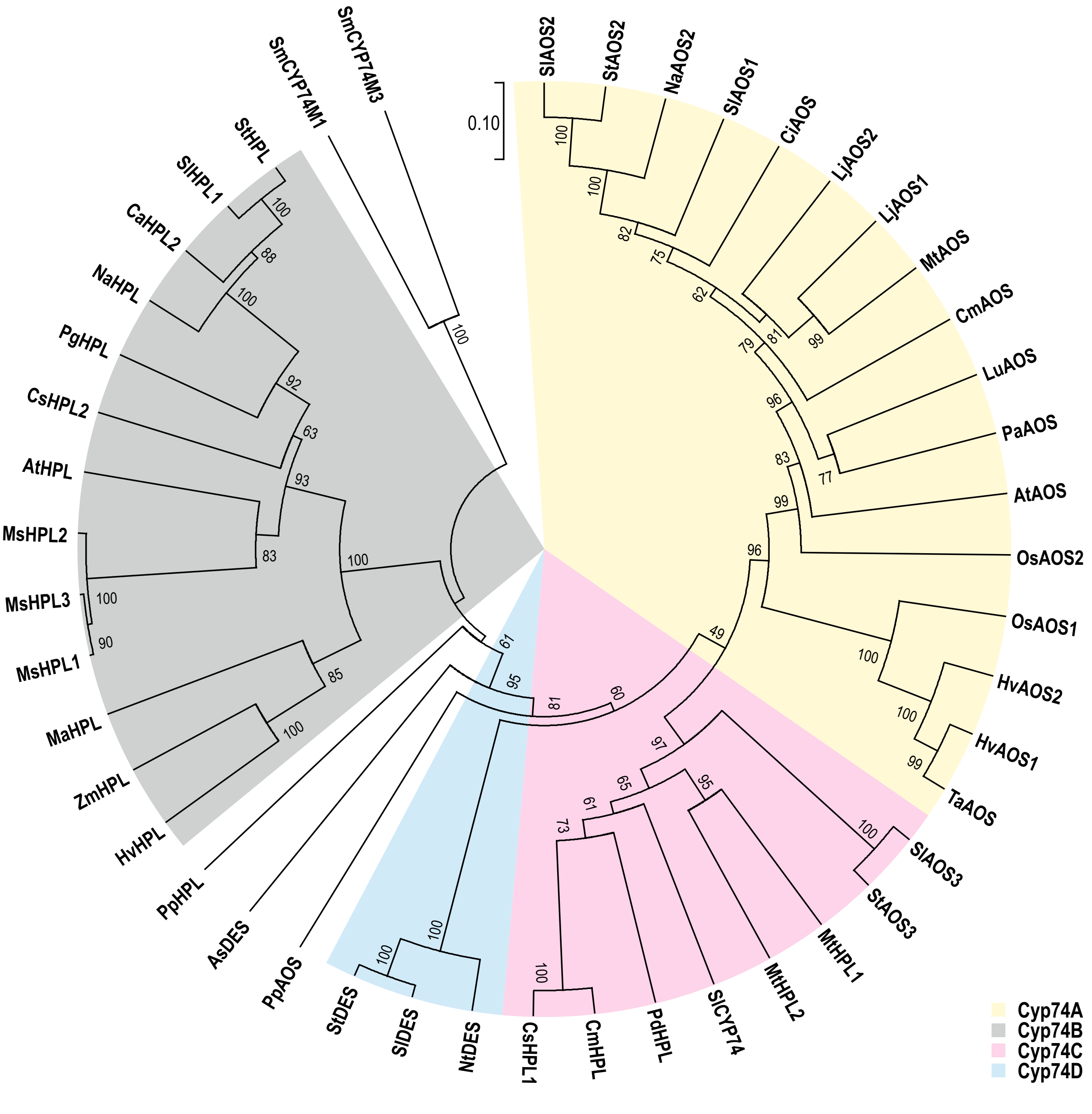
0.10
SlAOS2
StAOS2
NaAOS2
SlAOS1
CiAOS
LjAOS2
LjAOS1
MtAOS
CmAOS
LuAOS
PaAOS
AtAOS
OsAOS2
OsAOS1
HvAOS2
HvAOS1
TaAOS
SlAOS3
StAOS3
MtHPL1
MtHPL2
SlCYP74
PdHPL
CmHPL
CsHPL1
NtDES
SlDES
StDES
PpAOS
AsDES
PpHPL
HvHPL
ZmHPL
MaHPL
MsHPL1
MsHPL3
MsHPL2
AtHPL
CsHPL2
PgHPL
NaHPL
CaHPL2
SlHPL1
StHPL
SmCYP74M1
SmCYP74M3
Cyp74A
Cyp74B
Cyp74C
Cyp74D
